# Interpersonal Biofeedback in Psychodynamic Psychotherapy

**DOI:** 10.3389/fpsyg.2020.01655

**Published:** 2020-08-04

**Authors:** Johann Roland Kleinbub, Stefania Mannarini, Arianna Palmieri

**Affiliations:** ^1^Department of Philosophy, Sociology, Education, and Applied Psychology, Section of Applied Psychology, University of Padova, Padova, Italy; ^2^Interdepartmental Center for Family Research, University of Padova, Padova, Italy; ^3^Padova Neuroscience Center, University of Padova, Padova, Italy

**Keywords:** interpersonal biofeedback, physiological synchronization, psychodynamic psychotherapy, embodied-cognition, attachment, empathy

The idea that the patient-therapist relationship is at the core of change in psychotherapy has found broad consensus and ample empirical evidence in recent years (Norcross and Wampold, 2011). Engaging in an efficacious relationship is part of being an expert therapist (Kramer and Stiles, 2015), and while specific techniques are easily learnable, managing to “read the room” and to flexibly modulate the therapeutic response is still more an art than a science (McWilliams, 2004). With this article, we present the theoretical foundations supporting the development of “interpersonal biofeedback” as a tool to enhance therapists' awareness of unconscious interpersonal regulation dynamics.

## Relationship as Interpersonal Regulation

In psychodynamic theory, the therapeutic relationship has been studied through various constructs, such as transference-countertransference (Racker, 1982), therapeutic alliance (Safran, 2003), and intersubjectivity (Mitchell, 2000), each one marked by a plurality of descriptions, developments, and integration attempts. Broadly speaking, though, the psychodynamic views of this relationship shifted across the years from something that “happens within” to something that “happens between” patient and therapist. This idea was influenced by the development of the general systems theory and its idea that every human relationship can be modeled in terms of mutual regulation between interacting sub-systems (Gelso and Hayes, 1998). This conception, indeed, emerges in the writing of influential contemporary authors in the field. For instance, Safran and Muran (2006) argue that the construct of alliance as a “static variable that is necessary for the therapeutic intervention to work” should be superseded by the conception of a “constantly shifting, emergent property of the therapeutic relationship.” Furthermore, the authors suggest that instead of a collaboration, the patient- therapist interaction should be understood as an “ongoing process of negotiation,” further stressing the co-regulatory nature of the process.

Similarly, Kramer and Stiles (2015) defined the clinical relationship in terms of therapist responsiveness, i.e., the ability to respond to patients' requirements and characteristics as they emerge in the therapy process. Responsiveness is a “generic and ubiquitous principle of interpersonal regulation and attunement,” and an attitude that is prescribed by most therapeutic approaches, even, paradoxically, in strictly codified intervention manuals (Stiles et al., 1998). Furthermore, the right intervention at the wrong time may not yield a positive outcome. Considering relationship in terms of appropriate responsiveness or regulation introduces a temporal dimension in the study of clinical techniques, which will be crucial for the current proposal.

## Relationships are Embodied

An efficacious interpersonal regulation is not only a matter of choosing the right words. As in each human relationship, patient-therapist interactions involve the exchange of non-verbal, affective, and generally unconscious information. Unconscious communication emerges out of multi-layered, sensory-emotional, and enacted experiences, as described by the field of embodied-cognition (Shapiro, 2012; Barsalou, 2016). Such a notion, in extreme synthesis, overcomes mind-body dualities by drawing from the phenomenological idea that any experience and knowledge of the world is mediated by sensorial perceptions. Through various mechanisms, which are being validated by neuroscientific findings (e.g., Friston, 2009), the brain clusters these sensory-motor perceptions in “embodied representations.” These act both as heuristic prototypes, molding new perceptions, and as the neuronal substrates of internal objects, enabling secondary cognition.

Embodied-cognition was already present in its earliest stages in Freud's work: “The ego is first and foremost a bodily ego; it is not merely a surface entity, but is itself the projection of a surface” (Freud, 1923, p. 26), meaning that the ego is ultimately derived from bodily sensations. This embodied perspective remained at the core of psychodynamic thinking (see Fonagy and Target, 2007), and especially in its most relational facets, such as empathy and attachment (Mannarini et al., 2017). In the next paragraphs we present these two examples, where theory was able to intertwine with emerging neuroscientific and ethologic discoveries, demonstrating the embodied nature of interpersonal regulation.

### Embodied Empathy

The theorization of empathy developed together with psychoanalytic theory, initially thought of as a form of identification (Freud, 1920; Reik, 2013). Later authors, such as Kohut, Schafer, and others (Levy, 1985), further helped connote the psychodynamic understanding of the concept. Empathy is described as the core mechanism through which symbolic, somatic, and affective information is shared, consciously and unconsciously, between the therapeutic dyad, enabling both mutual understanding and transferential dynamics (Levy, 1985). Citing Freud (1912), the analyst “must turn his own unconscious like a receptive organ toward the transmitting unconscious of the patient.”

Modern neuroscience, notably through the discovery of mirror neurons (for a review see Jeon and Lee, 2018), confirmed many of these early intuitions on empathy (Decety et al., 2012).

After two decades of study, leading scholars in the field, such as de Waal and Preston (2017), concluded that “the emotional states of others are understood through personal, embodied representations that allow empathy and accuracy to increase based on the observer's past experiences.” Similarly, Rizzolatti and Caruana (2017) write that “empathy is based on personal, embodied representations of emotions that are mediated by the mirror mechanism.”

In other words, our physical and emotional experiences, such as joy, fear, feeling nurtured, hunger, etc., are “recorded” in our brains, and those “recordings” are immediately and unconsciously reactivated once we perceive similar experiences occurring in another person. Crucially, these re-activations do not involve isolated brain areas, but entire brain networks involving the processing of somatosensorial, emotional, and verbal experiences, as well as mnestic and higher cognitive functions (de Waal and Preston, 2017).

Furthermore, the enactment of these representations is not limited to the central nervous system. Through the Central Autonomic Network (CAN), activations of brain areas (namely the anterior cingulate, ventromedial prefrontal cortex, insular cortex, amygdala, and hypothalamus) modulate the activity of the autonomic nervous system, regulating the physiology throughout the whole body (Benarroch, 1993). The connecting function of CAN suggests that it may be possible to indirectly study the functioning of empathy through psychophysiological techniques (Ramachandra et al., 2009). Indeed, various authors managed to predict or measure empathy by studying the interpersonal regulation of physiological activity, or “physiological synchronization,” of patient-therapist (Marci et al., 2007; Kleinbub et al., 2012, 2019; Messina et al., 2013; Kleinbub, 2017; Wiltshire et al., 2020) and non-clinical dyads (Palumbo et al., 2017). Physiological synchronization, being associated with empathy, may thus be interpreted as the reflection of central mirror activity (Palmieri et al., 2018a) and offer the possibility of measuring interpersonal regulation of unconscious emotions on a moment-to-moment basis.

### Embodied Attachment

A similar reasoning can be drawn for attachment theory. Its modern developments describe characteristic patterns of cognition or, specifically, “states of mind that regulate attention with respect to perceptions or memories of attachment” with the primary object (Main et al., 1985). In discussing the mechanisms through which continuity in attachment between infancy and adulthood is created, Fonagy and Target (2007) suggest that physical and bodily experiences in children are stored as unconscious meanings (or, we shall say, embodied representations) which are enacted in adult communication through the metaphoric use of syntax, prosody, and phonation. Indeed, as first described by Mary Main (Hesse, 2008), different attachment classifications predict distinct narrative structures in adults, consisting of specific adherences or violations of Grice's assumptions of cooperative conversation, one of the basic tenets of pragmatics. It is important to note that linguistic characteristics associated with attachment classifications have been observed even when people were communicating about non-attachment related topics or interlocutors (Talia et al., 2017), highlighting how early experiences deeply shape not only the way we enact intimate relationships, but also the general way we communicate. This becomes of paramount importance in therapy where, as previously discussed, the negotiation of symbolic contents by means of verbal and non-verbal communication is the key to a successful intervention. Indeed, research showed that patients' Adult Attachment Interviews predicted their experience, representation, and communication about the relationship with their therapists (Talia et al., 2019), and that patients' transcripts characterized by secure narratives were associated with more rupture repairs and the ability to repair more intense ruptures (Miller-Bottome et al., 2019).

The role of embodied representations in attachment, though, is not limited to a vague influence of early experiences on adult's “higher” cognitive processes. Indeed, the enactment of those representations in everyday relationships directly influences a broad range of neural, physiological, and hormonal regulation processes (Insel, 2000; Diamond, 2001), and, crucially, the way these regulations are negotiated during interaction with others. In a recent study, Palmieri et al. (2018b) showed how manipulating attachment representations in therapists could alter their physiological synchronization with patients in a simulated intake interview. Furthermore, preliminary evidence (Kleinbub et al., 2020) showed that moment-by-moment physiological synchronization could be linked to specific verbal processes. In that study, we reported clinical vignettes where the temporal dynamics of physiological synchronization matched the coding of the therapy transcript through the Patient Attachment Coding System (PACS; Talia et al., 2017). Specifically, we observed that prosodic markers associated with secure attachment occurred simultaneously to sudden increases in skin conductance synchrony, and that those high synchronization phases lasted for the whole patient-therapist exchange, until the topic was changed.

## Interpersonal Biofeedback

Through these examples, we aimed to show recent research efforts, linking the embodied nature of interpersonal regulation processes to efficacious therapeutic relationship. Beyond just showing a link between mind and body, these studies show how bodily regulation plays an active role in these mechanisms, offering researchers an objective way to measure emotional and autonomic responses to conscious and unconscious stimuli through physiological measurements. But if these claims are true, and the empirical results stand the test of the necessary replications, the question becomes: can we use this knowledge and technology to aid and enhance clinical practice? An enlightening example is discussed in a case study by Marci and Riess (2005), where interpersonal physiology enhanced the exploration of conscious and unconscious processes. The skin conductance data allowed the therapist to acknowledge the high level of anxiety in the patient, which was hidden by a calm and reserved presentation.

“In the best of all possible worlds,” wrote (Chused, 1991), “an analyst is sensitive to his patient's transference, as expressed in either words or action, but does not act,” meaning that ideally therapists should be aware of the non-verbal and affective levels of negotiation with patients. Often though, this awareness is only achieved *a posteriori*, after an unconscious reaction happened in the therapist, leading to an observable behavior, response, or attitude (Sandler, 1976), or even in much later sessions or during supervision. What if we could provide this awareness, or at least a part of it, through moment-by-moment interpersonal physiological measures? Indeed, these measures have been shown to assess features of empathy, attachment, and alliance, information which could easily be provided in real-time to therapists, enriching their awareness of themselves and their patients. Such a tool could help therapists of any experience level and training (e.g., Gennaro et al., 2019) to get nearer to Chused's “best of all possible worlds.”

**Figure 1:**
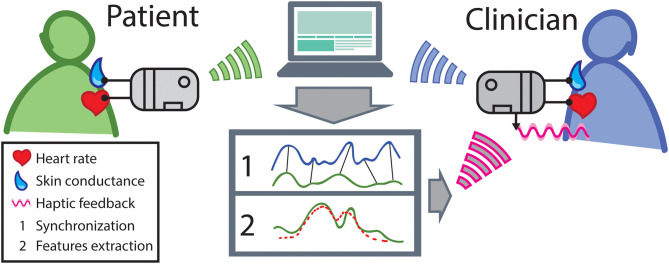
A hypothetical implementation of interpersonal biofeedback setup. Other types of sensors could be used as well, such as respiration bands, temperature sensors, electromyography, etc.

In Figure 1, we propose a schematic representation of how such an interpersonal biofeedback system could work. Heart rate and skin conductance signals^1^ would be simultaneously acquired in both patient and therapist through a wireless wearable device and sent to a PC or smartphone. Here the signals would be processed to: (1) assess the continuous degree of physiological synchronization, in order to detect phases of high and low attunement; and (2) extract salient features, such as markers of specific processes (e.g., secure-attachment communication or alliance ruptures), specific patient emotions (for a review see Shu et al., 2018), or sudden transitions of temporal dynamics (e.g., from a calm to an excited state). This information would then be transmitted back to the therapist's device and communicated to him by means of haptic signals.

### Roadmap

Interpersonal biofeedback is still a concept, and developing a working implementation represents a challenge requiring multiple research efforts. We identified three domains that need to be explored to achieve this goal.

#### Identification of Processes

Most existing studies investigated synchronization and empathy, outcome, etc., only at a correlational level (Kleinbub et al., 2020). Further studies of moment-by-moment physiological activity and synchronization in therapy are needed, in order to recognize which clinical processes can be identified with adequate precision, and through which combination of signals.

#### Feedback Modulation

Good biofeedback implementations allow implicit learning (Gaume et al., 2016), which represents a twofold advantage. First, therapists would be able to use the tool without an in-depth knowledge of its theoretical assumptions, and second, its use would only slightly impact therapists' cognitive load. For this to happen, though, future research should focus on how to transform the complex dyadic information extracted from multiple physiological signals into a feed of information that is intuitive and easy to understand for the therapist.

#### Validation

As a tool that directly influences therapy, assessing interpersonal biofeedback safety, validity, and efficacy will be of uttermost importance. According to established guidelines (Moss and Gunkelman, 2002), a biofeedback intervention can be considered efficacious after replication in at least two independent high-quality randomized control trials, possibly including a placebo comparison. Preliminary steps, though, may involve pilot studies on non-clinical interactions and in-depth interviews with experienced therapists.

## Conclusion

While therapist's sensitivity and intuition will long remain the fundamental tools of the trade, we believe that these traits can, should, and will be augmented by neuroscientific and technological advancements. Furthermore, such a paradigmatic challenge might prove to be a terrific opportunity for the psychodynamic movement. In light of the recent call for evidence-based treatments, psychoanalysis and psychodynamic psychology are suffering a continuous decline in relevance and prestige (e.g., Paris, 2017). Yet, no other clinical approach can boast such a deep and refined comprehension of intersubjectivity and interpersonal regulation. Psychodynamic theory, through its strong ties with embodied-cognition (Fonagy and Target, 2007) and dynamic-systems (Gelo and Salvatore, 2016), provides the most natural framework for the development and application of a tool aimed to make unconscious interpersonal regulation explicit.

In conclusion, interpersonal biofeedback could represent a powerful tool for psychodynamic psychotherapy, providing a new “sense” to therapists of any level of expertise. This augmented perception could enhance their awareness of unconscious interpersonal regulation dynamics on a moment-to-moment basis. Indeed, physiology offers a deeper look into affective dynamics than observable behavior, one that is often deeper than even self-awareness.

Jung once notably said that physiology is a looking glass into the unconscious (Brown, 1977). Through interpersonal biofeedback techniques, we may provide dynamic therapists with a way to access this looking glass in real-time during therapy sessions, to the great benefit of patients.

## Author Contributions

All authors provided substantial contributions to the conception of the work, provided critical revisions to the manuscript, and approved the last version for publication.

## Conflict of Interest

The authors declare that the research was conducted in the absence of any commercial or financial relationships that could be construed as a potential conflict of interest.

## Footnotes

^1^While many other physiological and non-physiological (Wiltshire et al., 2020) signals have been used to assess interpersonal synchronization, skin conductance and heart rate provide distinct advantages, such as the relative ease of acquisition and well-studied relationship with emotion regulation. Nonetheless, other types of signals may be used.
